# Overexpression of NK4 gene in TU212 affects migratory activity in laryngeal squamous cell carcinoma

**DOI:** 10.3389/fonc.2025.1553626

**Published:** 2025-08-01

**Authors:** Yixuan Huo, Wei Zhang, Fan Yang, Wenhua Shao, Guozheng Cong, Shoukai Zhang

**Affiliations:** 1 First Clinical Medical College, Ningxia Medical University, Yinchuan, China; 2 Lanzhou University, Lanzhou, Gansu, China; 3 Lanzhou Veterinary Research Institute, Chinese Academy of Agricultural Sciences, Lanzhou, Gansu, China; 4 Gansu Provincial Hospital, Lanzhou, Gansu, China

**Keywords:** apoptosis, gene expression, laryngeal squamous cell carcinoma, NK4, RNA-seq, migration

## Abstract

**Background:**

Abnormal activation of the hepatocyte growth factor (HGF) and c-mesenchymal–epithelial transition factor (c-Met) signaling pathway is associated with tumor occurrence and development. Serum HGF concentrations are significantly higher in patients with advanced and poorly differentiated laryngeal squamous cell carcinoma than those with early and highly differentiated disease. *NK4*, a splice variant of HGF, can competitively bind to c-Met and acts as a specific antagonist of HGF. Although preliminary research has been conducted on the tumor-suppressing function of the *NK4* gene, its specific mechanism of action in laryngeal cancer remains unclear.

**Methods:**

Stable laryngeal squamous cell carcinoma cell lines expressing *NK4* were developed using a lentiviral packaging method. The experimental group was labeled with PLV-NK4-TU212, whereas the control group was labeled with PLV-NC-TU212. Western blotting verified a stable expression. The functions of the *NK4* molecule were assessed using MTT, EMT, and apoptosis assays, and cell lines were subjected to transcriptome sequencing.

**Results:**

Protein expression analysis showed that *NK4* was stably expressed. Compared with the wild-type and negative control groups, overexpression of the *NK4* gene inhibited the migration and proliferation of laryngeal squamous cell carcinoma cells and induced cell apoptosis. Transcriptome sequencing revealed that the expression levels of 320 genes differed significantly, with 189 upregulated and 131 downregulated genes.

**Conclusion:**

In this study, a TU212 laryngeal squamous cell carcinoma cell line overexpressing *NK4* was constructed using a lentiviral packaging system. Functional experiments showed that PLV-NK4-TU212 cells exhibited a significantly reduced migration rate, decreased proliferative ability, and increased apoptosis rate. The results of this study provide an experimental basis for *NK4* as a potential therapeutic target for laryngeal squamous cell carcinoma highlighting its translational medical value.

## 1 Introduction

According to global epidemiological data, laryngeal cancer, a major type of malignant head and neck tumor, accounts for approximately 1% of all malignant tumors (1). Approximately 90% of the pathological types are squamous cell carcinomas (2), and malignant transformation originates from the abnormal proliferation of laryngeal mucosal epithelial cells (3). Despite the multidisciplinary integration of innovative therapies in recent years, such as surgical resection, radiotherapy, sensitization chemotherapy, and PD-1/PD-L1 immune checkpoint inhibitors, the incidence and mortality rates are increasing (4). Laser minimally invasive surgery (TLM) for early-stage patients can effectively remove tumors while preserving laryngeal function as much as possible (5). In surgery combined with radiotherapy (6), chemotherapy, and targeted therapy, the 5-year survival rate for early-stage laryngeal cancer (T1–T2) is approximately 80%, while the 5-year survival rate for locally advanced laryngeal cancer (T3–T4) is approximately 50% (7, 8).

This clinical dilemma is primarily attributable to the unique molecular and pathological characteristics of laryngeal cancer. LSCC is prone to local infiltration, cervical lymph node metastasis, and chemotherapy resistance (9). To date, no accurate biomarkers have been determined for treatment response in LSCC. Therefore, exploring the molecular regulatory network of invasion and metastasis in LSCC may provide a theoretical basis for the development of new strategies for targeted therapy.

Clinical studies have shown that the aberrant activation of the hepatocyte growth factor (HGF)/c-Met signaling pathway is associated with tumorigenesis and progression. Met, a receptor for HGF, is a proto-oncogene product of a heterodimeric tyrosine kinase (10). After binding to HGF, it initiates downstream signaling pathways, such as MAPK, STAT, PI3K, and NF-κB, to regulate cell proliferation, invasion, and migration (11). Serum HGF concentrations in patients with advanced and poorly differentiated laryngeal squamous cell carcinoma are significantly higher than those in patients with early stage and highly differentiated disease (12). Studies have shown that *NK4*, an HGF antagonist, inhibits HGF/c-Met-induced tumor growth, metastasis, and invasion antagonism ultimately leading to apoptosis. *NK4* consists of a hairpin structural domain at the N-terminal end of the α-chain of HGF and four Kringle regions, which is obtained from the cleavage of HGF by proteolytic digestion. *NK4*, a splice variant of HGF, competes with c-Met for binding and is a specific antagonist of HGF (13). An adenoviral vector carrying a stably expressed *NK4* gene was found to inhibit the growth of a bone metastatic tumor model and prolonged the survival of mice (14). Human placental-derived mesenchymal stem cells expressing *NK4* (PDMSC-NK4) have been reported to exert antitumor effects on glioblastoma cells (15). Moreover, NK4 inhibits cancer growth *in vivo* by suppressing IDO expression in tumors and promoting NK cell accumulation (16).

Lentiviruses can efficiently deliver target genes into tumor cells and stably integrate them into the host cell genome to achieve long-term and stable gene expression, thereby aiding in the genetic modification of tumor cells and the study of regulatory gene functions. Using a lentiviral packaging system to construct cell lines that stably express genes is a powerful tool for gene therapy and mechanistic research on tumors. For instance, localized STAT3 knockdown in the brain, achieved via lentiviral vectors encoding STAT3 shRNA, was established, and the NLRP3-STAT3 interaction was validated. This model system was then utilized to determine if JAK2 inhibition confers neuroprotection following ischemic stroke (17). Furthemore, previous research has also reported a lentiviral overexpression vector co-expressing the CopGFP and PuroR reporter genes was successfully engineered. Validation in hepatocellular carcinoma (HCC) cells demonstrated its efficacy in promoting the expression of a target gene with an extended coding sequence (18). Lentivirus-mediated gene transfer was employed to establish a stable cell line expressing the αvβ3 integrin, which exhibited enhanced susceptibility to foot-and-mouth disease virus (FMDV). This model provides a valuable tool for investigating αvβ3 integrin functions (19). Based on this literature, we used lentiviral packaging technology to construct a TU212 cell line of laryngeal squamous cell carcinoma overexpressing *NK4* and verified its biological functions. The aim of this study was to investigate the function and mechanism of action of *NK4*, providing a theoretical basis for assessing its role in the progression of laryngeal cancer, as well as its potential therapeutic value and its clinical application.

## 2 Materials and methods

### 2.1 Cells, plasmids, and viruses

The TU212 cell line was purchased from Beina and cultured in RPMI-1640 medium supplemented with 10% fetal bovine serum and 1% antibiotics at 37°C in a 5% CO2 incubator. PLV-NK4-Flag plasmid was constructed by the research team in the earlier stage. Human embryonic kidney cells (HEK-293T), psPAX2 (Addgene, 12260), and pMD2.G (Addgene, 12259) were generously provided by the Lanzhou Veterinary Research Institute of the Chinese Academy of Agricultural Sciences.

### 2.2 Reagents and antibodies

RPMI 1640 cell culture medium (Gibco) and 0.25% EDTA (Gibco) trypsin were purchased from Gansu Pengcheng Biotechnology Development Co. Ltd. Fetal bovine serum (FBS) was purchased from Guangzhou Lucheng Biotechnology Co. Ltd. Polyplus jet PRIME transfection reagent was purchased from Gene Biotechnology Co. Ltd. Western antibody dilution buffer was purchased from Biotime Biotechnology Co. Ltd. Mouse anti-Flag antibody was purchased from Sigma-Aldrich (Shanghai) Trading Co. Ltd., and the protein pre-stained marker was purchased from Invitrogen.

### 2.3 Construction and validation of NK4-overexpressing laryngeal squamous cell carcinoma TU212 cell line

**Figure 1:**
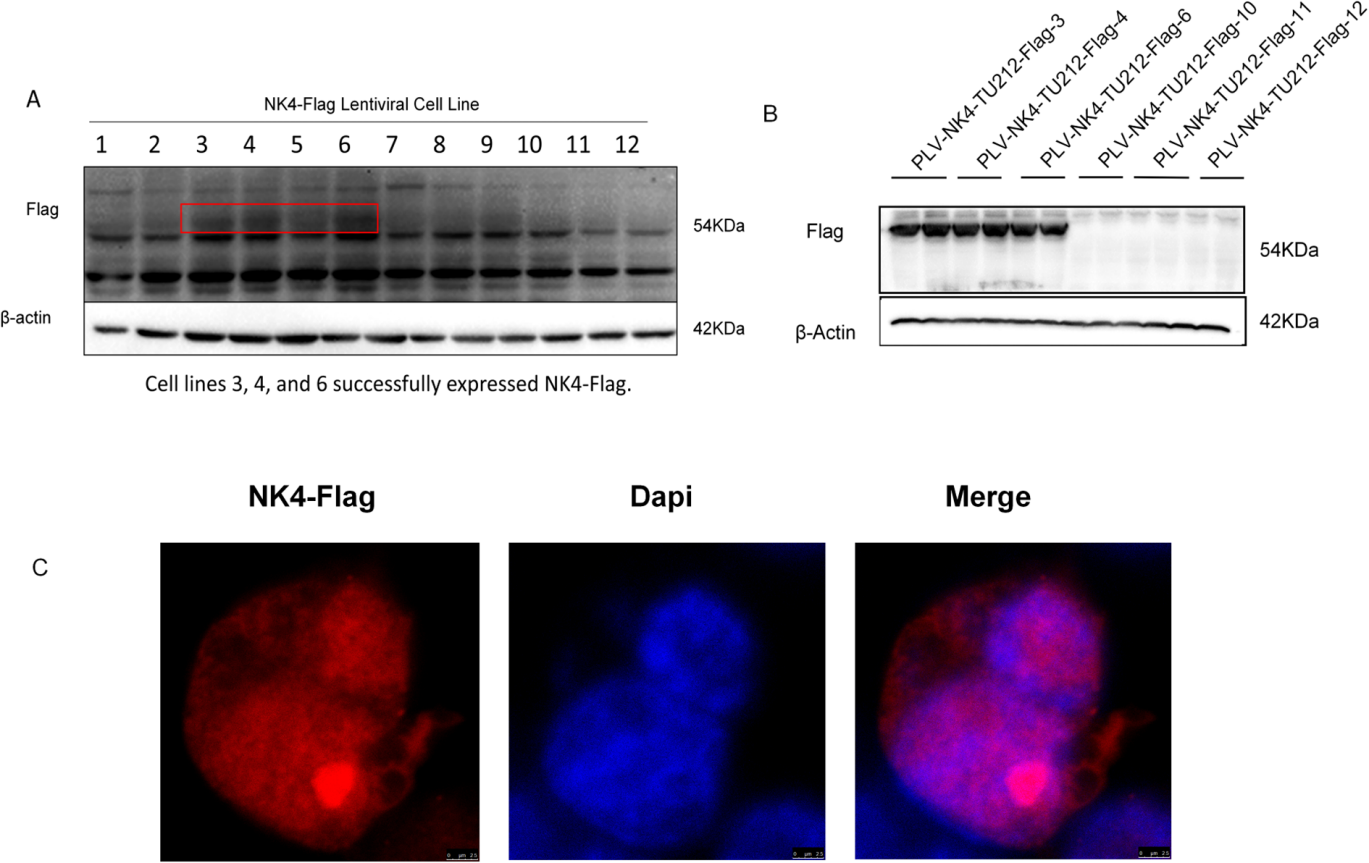
Construction and validation of NK4-overexpressing laryngeal squamous cell carcinoma TU212 cell line. **(A)** In the first-generation cell line screening, 3, 4 and 6 demonstrated successful expression of NK4-Flag, while 10,11, and 12 showed no detectable NK4-Flag expression. **(B)** Fifth generation cell line. **(C)***NK4* gene is stably expressed and localized in the nucleus.

After reviving HEK-293T cells to the second generation, the cells were found to be in good condition and seeded at a density of 5 × 10^6^ cells in a 100-mm culture dish. When the cell density reached approximately 70%, 3 μg of pLV-puro-3×-Flag-JMJD6 plasmid, 2 μg of psPAX2 packaging plasmid (Addgene, 12260), and 1 μg of pMD2.G envelope plasmid (Addgene, 12259) were transfected using jetPRIME transfection reagent. The supernatants containing the lentivirus were collected 24 and 48 h post-transfection, filtered for purification, and the packaged lentivirus was used to infect the TU212 cells. After 48 h, the cells were digested with trypsin, and the medium containing 10 μg/ml of puromycin was added for selection. The medium was changed after 24 h, and the medium containing 5 μg/ml of puromycin was added to continue selection. Finally, the surviving cells were subcloned using the limiting dilution method, and the selected monoclonal cells were expanded and cryopreserved. Cells cultured for five generations were mixed with loading buffer, and 20 μl of the protein sample was used for SDS-PAGE. After electrophoresis, the proteins were transferred onto a nitrocellulose membrane at a constant voltage of 100 V. The membrane was blocked at room temperature for 2 h with 5% non-fat milk solution prepared in TBST followed by overnight incubation at 4 C with the corresponding primary antibody. After washing with TBST, the secondary antibody was added and incubated at room temperature for 2 h. Finally, an ECL chemiluminescent solution was used for fully automated chemiluminescent imaging (**Figures 1A, B**). Cells that were continuously passaged for 10 generations were subjected to indirect immunofluorescence analysis. The results were observed using a laser confocal microscope (×60) (**Figure 1C**).

### 2.4 Functional validation of laryngeal squamous cell carcinoma cell lines stably expressing NK4

#### 2.4.1 The effect of PLV-NK4-TU212 on cell migration and apoptosis

**Figure 2:**
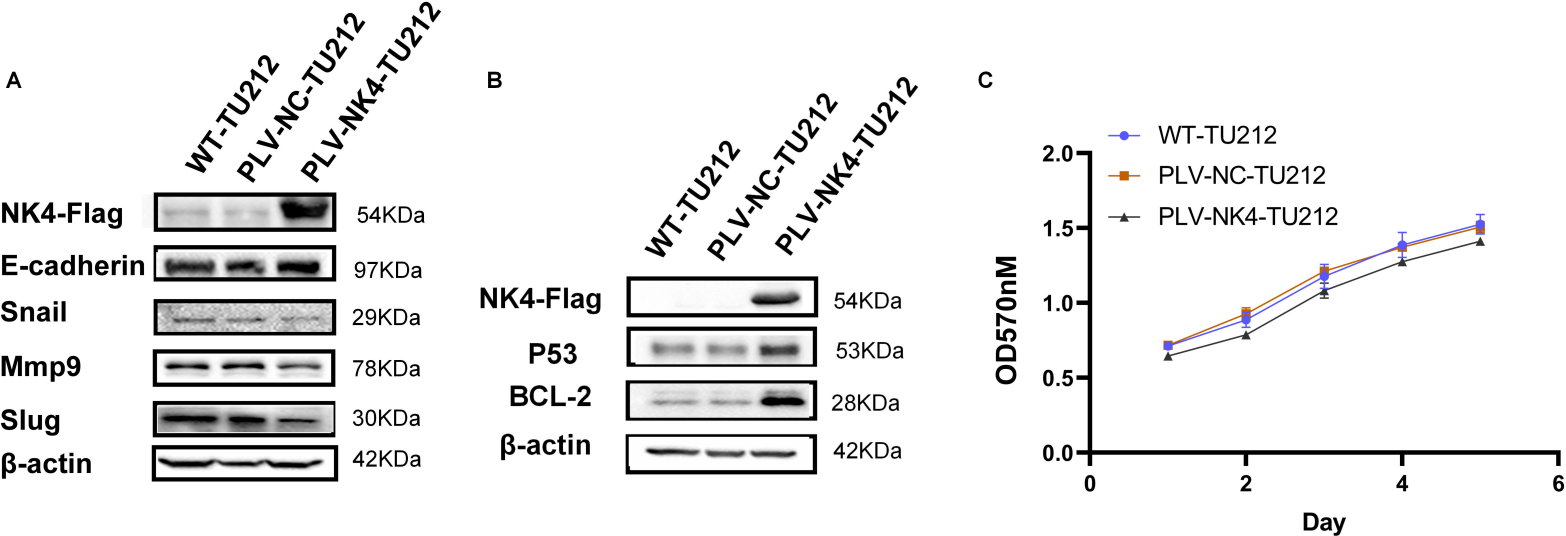
Functional validation of laryngeal squamous cell carcinoma cell lines stably expressing NK4. **(A)** Effect of overexpression of NK4 on TU212 cell migration. **(B)** Effect of NK4 overexpression on apoptosis in TU212 cells. **(C)** Effect of NK4 overexpression on the proliferation ability of TU212.

Western blot analysis was performed to detect EMT markers (E-cadherin, Snail, MMP9, and Slug) for PLV-NK4-TU212, with β-actin as the internal control (**Figure 2A**), and to show expression of P53 and Bcl-2 genes related to apoptosis (**Figure 2B**).

#### 2.4.2 MTT assay to detect the proliferative capacity of stably transformed cell lines

The cell concentration was adjusted to 2 × 10^4^ ml followed by inoculation into a 96-well plate and culturing for 24 h. The original culture medium was removed, the MTT solution was added, and the cells were incubated at a constant temperature for 4 h. The resulting supernatant was discarded before adding 100 μl of DMSO solution to each well. The cultures were shaken thoroughly for 10 min, and then the absorbance was measured at 570 nm. All cultures were continuously monitored for 7 days (**Figure 2C**).

#### 2.4.3 Scratch healing assay to detect the migratory ability of stabilized transient cell lines

**Figure 3:**
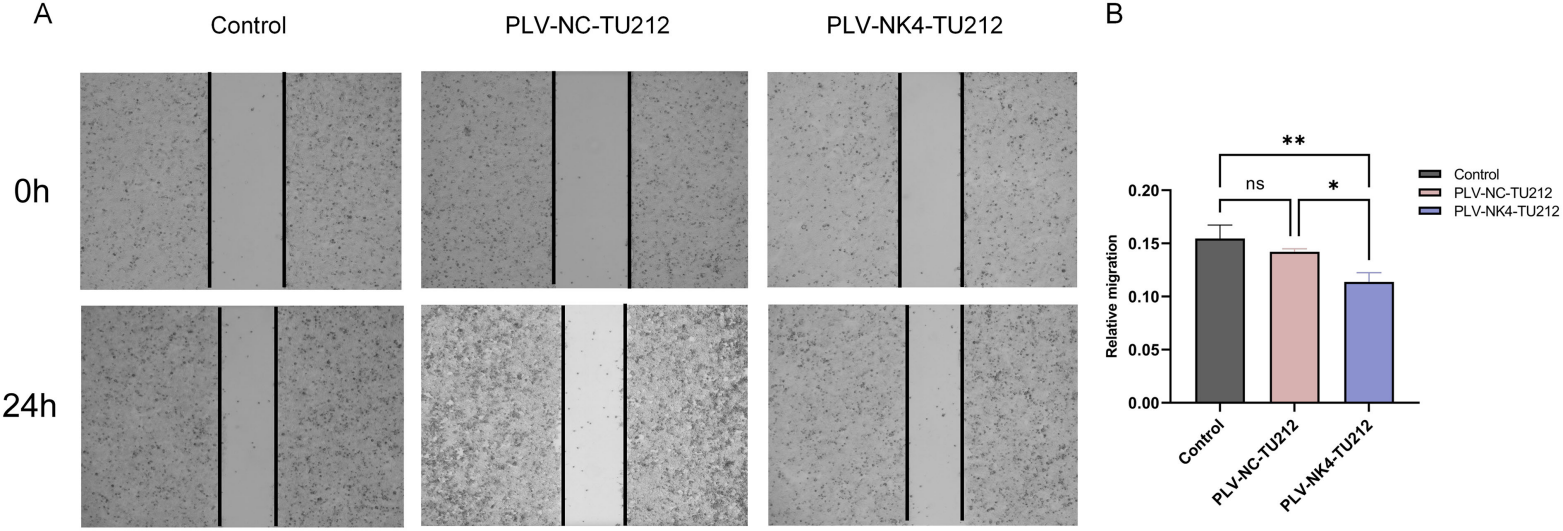
Scratch healing assay to detect the migratory ability of stabilized transient cell lines. **(A)** The wound area of TU212 cells was measured at 0 h and 24 h. **(B)** Quantification of cell migration. The effect of NK4 overexpression on the migratory ability of TU212 cells was examined using a wound healing assay. (*P<0.05; **P<0.01).

**Figure 4:**
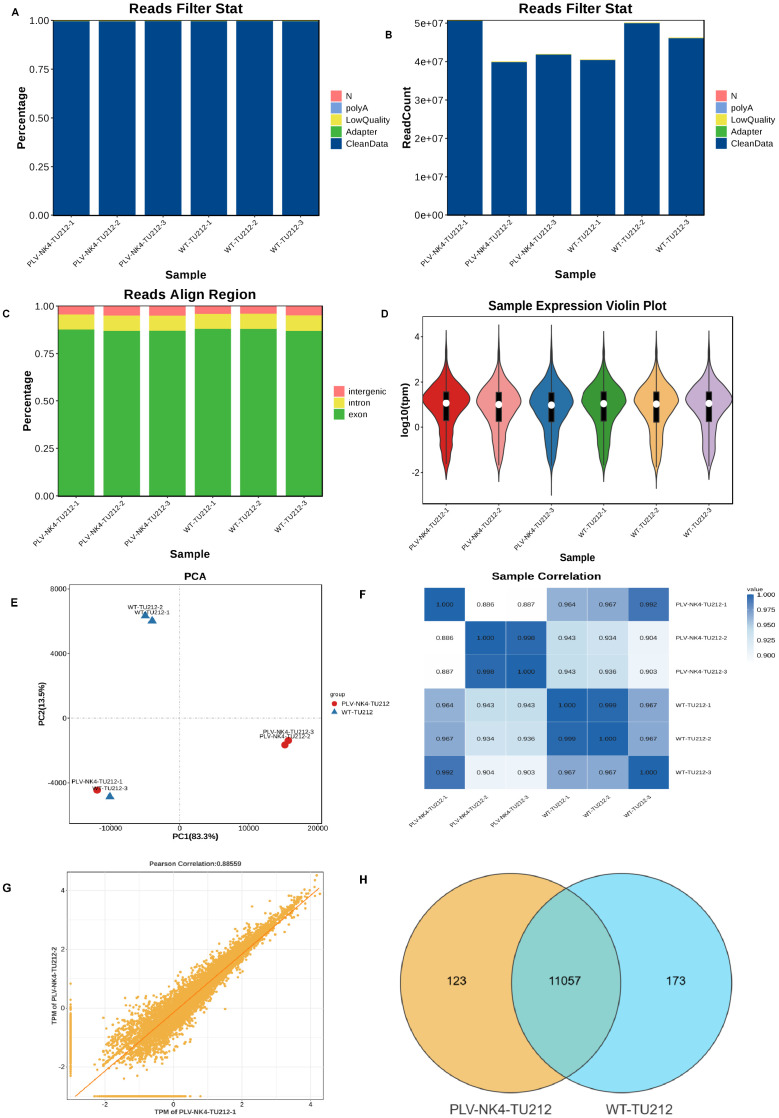
Data quality control, expression abundance distribution, sample PCA analysis, and correlation analysis. **(A)** Data preprocessing distribution chart (percentage), **(B)** Data preprocessing distribution chart (numerical), **(C)** Comparison reference area statistical chart, **(D)** Gene expression violin plot, **(E)** Sample Principal Component Analysis, **(F)** Sample Correlation Heatmap, **(G)** Sample Reproducibility Scatter Plot, and **(H)** Venn diagram.

Cells were seeded at a density of 2.5 × 106/mL in a six-well plate and cultured in a complete medium until 100% confluence was reached (**Figures 4A, B)**. A sterile pipette tip was used to create vertical scratches at the bottom of a six-well plate, which had been cultured in complete medium until reaching 100% confluence. The wells were then washed with PBS. Next, RPMI 1640 medium containing 2% serum was added to each well, and the plate was incubated at 37°C with 5% CO₂. Images were captured at 0 and 24 hours using an inverted light microscope. The scratch healing rate was calculated as follows: Scratch healing rate = [(0 h − 24 h) / 0 h] × 100% (**Figure 3**).

#### 2.4.4 Statistic analysis

Results are expressed as mean ± standard deviation. All data were analyzed using GraphPad Prism 9 (GraphPad Software, Inc., CA). Intergroup differences were assessed using Student’s t-test or unpaired two-tailed t-test. Two-way analysis of variance (ANOVA) was used for comparisons among multiple groups. A p-value <0.05 was considered statistically significant.

### 2.5 RNA-seq

#### 2.5.1 RNA sample preparation, library construction, and sequencing

The cells, WT-TU212 and PLV-NK4-TU212, were resuscitated and cultured in a medium containing 10% FBS and 1% dual antibiotics until the third generation. Total RNA was extracted using the TRIzol method, and eukaryotic mRNA was enriched with a poly (A) tail using magnetic beads with Oligo(dT) and then fragmented with a buffer. Using fragmented mRNA as a template and random oligonucleotides as primers, the first strand of cDNA was synthesized using the M-MuLV reverse transcriptase system. Subsequently, the RNA strand was degraded with RNase H, and the second strand of cDNA was synthesized in a DNA polymerase I system using dNTPs as raw materials. Purified double-stranded cDNA underwent end repair, A-tailing, and ligation of sequencing adapters. AMPure XP beads were used to select cDNA of approximately 200 bp, perform PCR amplification, and purify the PCR products again using AMPure XP beads to obtain the library and conduct quality control.

Clean data were obtained by removing adapters, polynucleotides, and low-quality sequences from the raw data. The total number of bases in the sample sequencing data was filtered to obtain a high-quality database (**Figures 4A, B**). The short-read alignment tool Bowtie2 (20) was used to align the clean reads to the ribosomal database of the species removing reads aligned to the ribosome without allowing mismatches. The remaining unmapped reads were used for subsequent transcriptome analysis. HISAT2 software was used to perform alignment analysis based on the reference genome. The distribution of reads in the exonic, intronic, and intergenic regions of the reference genome was determined based on the total mapped read alignment results (**Figure 4C**).

#### 2.5.2 Expression abundance distribution, sample PCA analysis, and correlation analysis

Based on the TPM values of each gene, the expression distribution of different sample genes or transcripts are displayed (**Figure 4D**). Principal component analysis was conducted using R (http://www.r-project.org/) (**Figure 4E**). The Pearson correlation coefficient between the expression levels of any two samples was calculated, and the correlation between any two samples was displayed in the form of a heatmap to examine the reproducibility among repeated samples within the test group (**Figure 4F**). The correlation coefficient between pairs of samples was calculated using the FPKM/TPM values of genes in the samples to assess the reproducibility of samples within the group (**Figure 4G**). Genes were selected based on gene abundance, and a Venn diagram was used to identify shared and unique genes between groups (**Figure 4H**).

#### 2.5.3 Differential gene analysis

**Figure 5:**
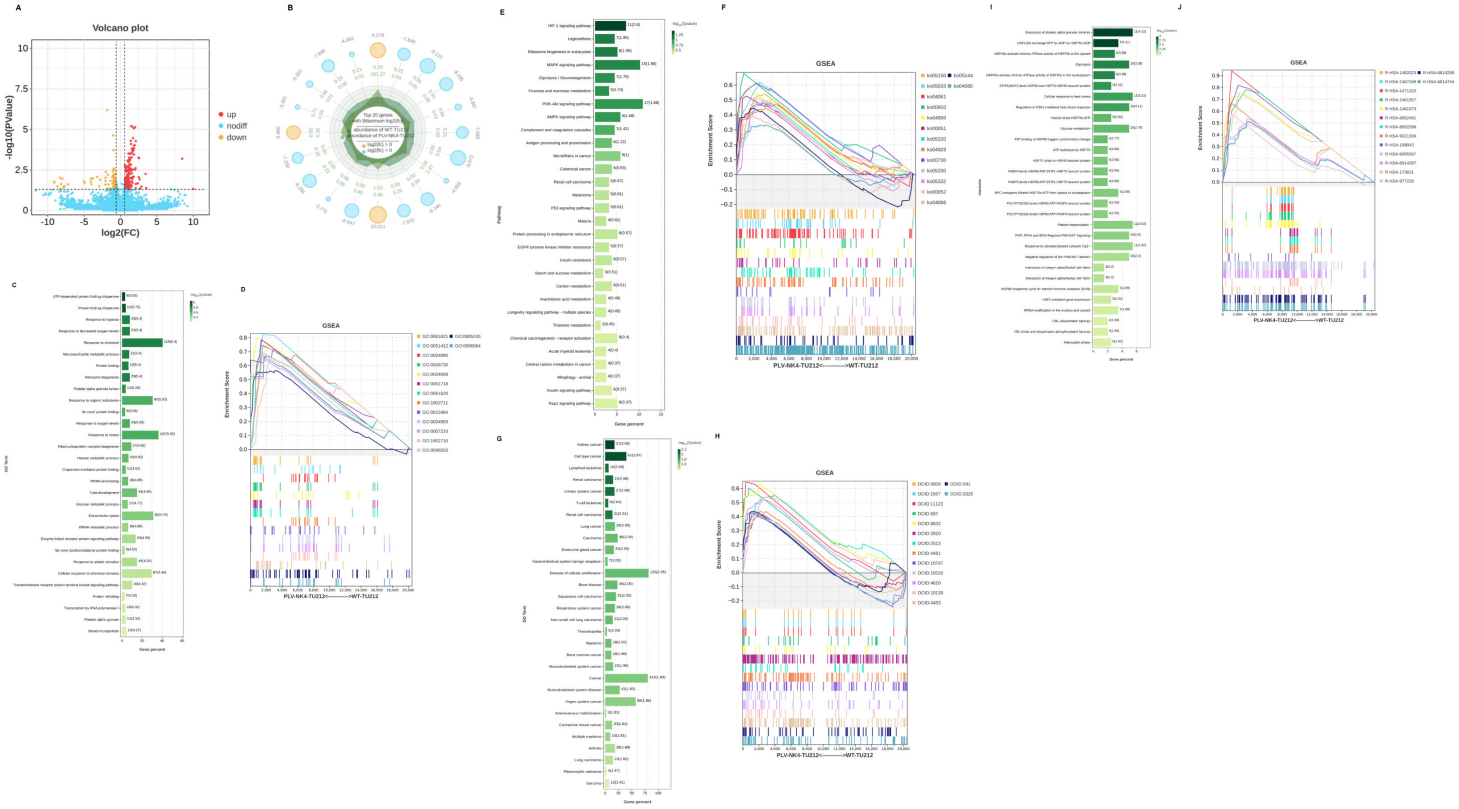
Differential gene analysis and GSEA analysis. **(A)** Differential basic analysis volcano plot, **(B)** Difference basic analysis radar chart, **(C)** GO enrichment, **(D)** GSEA-GO analysis, **(E)** KEGG enrichment, **(F)** GSEA-KEGG analysis, **(G)** DO enrichment, **(H)** GSEA-DO analysis, **(I)** Reactome enrichment, and **(J)** GSEA-Reactome analysis.

DESeq2 software was used to normalize the read count, calculate the probability of hypothesis testing (p-value) according to the model, and finally correct for multiple hypothesis testing to obtain the FDR value (false discovery rate), with the following conditions for difference screening: p-value < 0.05, |log_2_FC| > log_2_(1.5) (**Figures 5A, B**).

#### 2.5.4 GO and KEGG enrichment analysis

Differential genes were mapped to each term of the GO database (http://www.geneontology.org/), and the number of differential genes per term was calculated to obtain a list of differential genes with a certain GO function. The number of differential genes was used to generate statistics of GO entries that were significantly enriched in differential genes. KEGG is the main public database on pathways, which identifies the top biochemical metabolic pathways and signaling pathways in which differential genes are involved through significant pathway enrichment.

#### 2.5.5 DO and reactome enrichment analysis

DO (Disease Ontology) is a database that describes the relationship between gene functions and diseases. We map the differentially expressed genes to the terms in the DO database (http://disease-ontology.org/) and calculate the number of differentially expressed genes for each term resulting in a list of differentially expressed genes associated with a specific DO function and a count of these genes. We then apply hypergeometric testing to identify DO entries that are significantly enriched among the differentially expressed genes compared to the entire background.

The Reactome database gathers various reactions and biological pathways from several species. We mapped the differentially expressed genes to the various terms in the Reactome database (https://reactome.org/) and calculated the number of differentially expressed genes for each term resulting in a list of differentially expressed genes with a specific Reactome function and a count of these genes. We then applied hypergeometric testing to identify Reactome entries that are significantly enriched among the differentially expressed genes compared to the entire background.

#### 2.5.6 Transcription factor statistics and variable shear analysis

The transcription factor database (http://bioinfo.life.hust.edu.cn/AnimalTFDB4/#/) was used to annotate genes for transcription factors (TF), to determine whether a gene is a member of a specific transcription factor family, and summarize how many genes are in each TF family.

Alternative splicing (AS) is an important gene regulation mechanism in eukaryotes. We used rMATS to detect five types of alternative splicing events as follows: SE, RI, MXE, A5SS, and A3SS. We can perform differential AS analysis on samples with biological replicates.

## 3 Results

### 3.1 Identification of stable lentiviral cell lines expressing *NK4*

The lentiviral *NK4* overexpression cell lines selected under pressure with puromycin were passaged (**Figure 1A**), and fifth-generation cell lines were identified by western blotting (**Figure 1B**), as shown in **Figures 1A, B**. *NK4* was stably expressed in passaged cell lines 3, 4, and 6. The remaining cells were further cultured to the 10th generation, and the expression of the *NK4* gene in the cell lines was detected using an indirect immunofluorescence assay employing a mouse-derived anti-Flag tag antibody and an anti-488 fluorescent antibody as the primary and secondary antibodies, respectively, to verify the expression of the *NK4* gene in the cell lines. The results showed that *NK4* was stably expressed and localized in the nucleus.

### 3.2 Functional validation of laryngeal squamous cell carcinoma cell lines stably expressing *NK4*

#### 3.2.1 The effect of PLV-NK4-TU212 on cell migration and apoptosis

We performed Western blot analysis of several EMT (epithelial–mesenchymal transition) markers (E-cadherin, Snail, MMP9, and Slug) for cell line. The expression of the proteins Snail, MMP9, and Slug was decreased in PLV-NK4-TU212, which proved that overexpression of the *NK4* gene inhibited the process of EMT (**Figure 2A**). Immunoblotting showed that, compared to the control group, the expression of the apoptosis-related genes P53 and Bcl-2 was upregulated in PLV-NK4-TU212. These results indicate that NK4 gene overexpression induces apoptosis (**Figure 2B**).

#### 3.2.2 MTT assay to detect the proliferative capacity of stably transferred cell lines

The results of the MTT assay showed that the OD value of the PLV-NK4-TU212 group was significantly reduced compared with that of WT-TU212, and the OD value of the PLV-NK4-TU212 group was significantly reduced compared with that of the PLV-NC-TU212 group (**Figure 2C**). It indicates that overexpression of NK4 in laryngeal squamous cell carcinoma TU212 cell line inhibited cell proliferation.

#### 3.2.3 Scratch healing experiment

The scratch healing experiment tested the migration ability of stable cell lines, and the results showed that, compared to the PLV-NC-TU212 and WT-TU212 groups, the cell migration rate in the PLV-NK4-TU212 group decreased at 24 h suggesting that NK4 overexpression can inhibit the migration ability of TU212 cells.

#### 3.2.4 GO and KEGG enrichment analysis

We used RNA-seq to compare the gene expression profiles between PLV-NK4-TU212 and WT-TU212 cell lines, and the results showed that a total of 320 genes had significant differences in expression, of which 189 genes were upregulated and 131 genes were downregulated (**Figures 5A, B**). GO annotations were enriched in terms of molecular function, cellular component, and biological process. In the biological process, we mainly focused on cellular process, biological regulation, regulation of biological process, metabolic process, and response to stimulus; in the cellular component, we only focused on cellular anatomical entity and protein. anatomical entity and protein-containing complex. In terms of molecular function, binding and catalytic activity were differentially enriched (**Figure 5C**). KEGG enrichment analysis showed that among the top 20 significantly enriched pathways, they were mainly related to HIF-1, MAPK, and PI3K–AKt signaling pathways (**Figure 5E**).

#### 3.2.5 DO and Reactome enrichment analysis

The significant enrichment of organ system cancer and cell type cancer in the Disease Ontology (DO) enrichment analysis of differentially expressed genes suggests a dual regulatory mechanism of differentially expressed genes in cancer occurrence (**Figure 5G**). Differentially expressed genes are enriched in the hemostasis pathway, and their abnormalities are closely related to the process of tumor metastasis (**Figure 5I**).

#### 3.2.6 Transcription factor statistics and variable shear analysis

**Figure 6:**
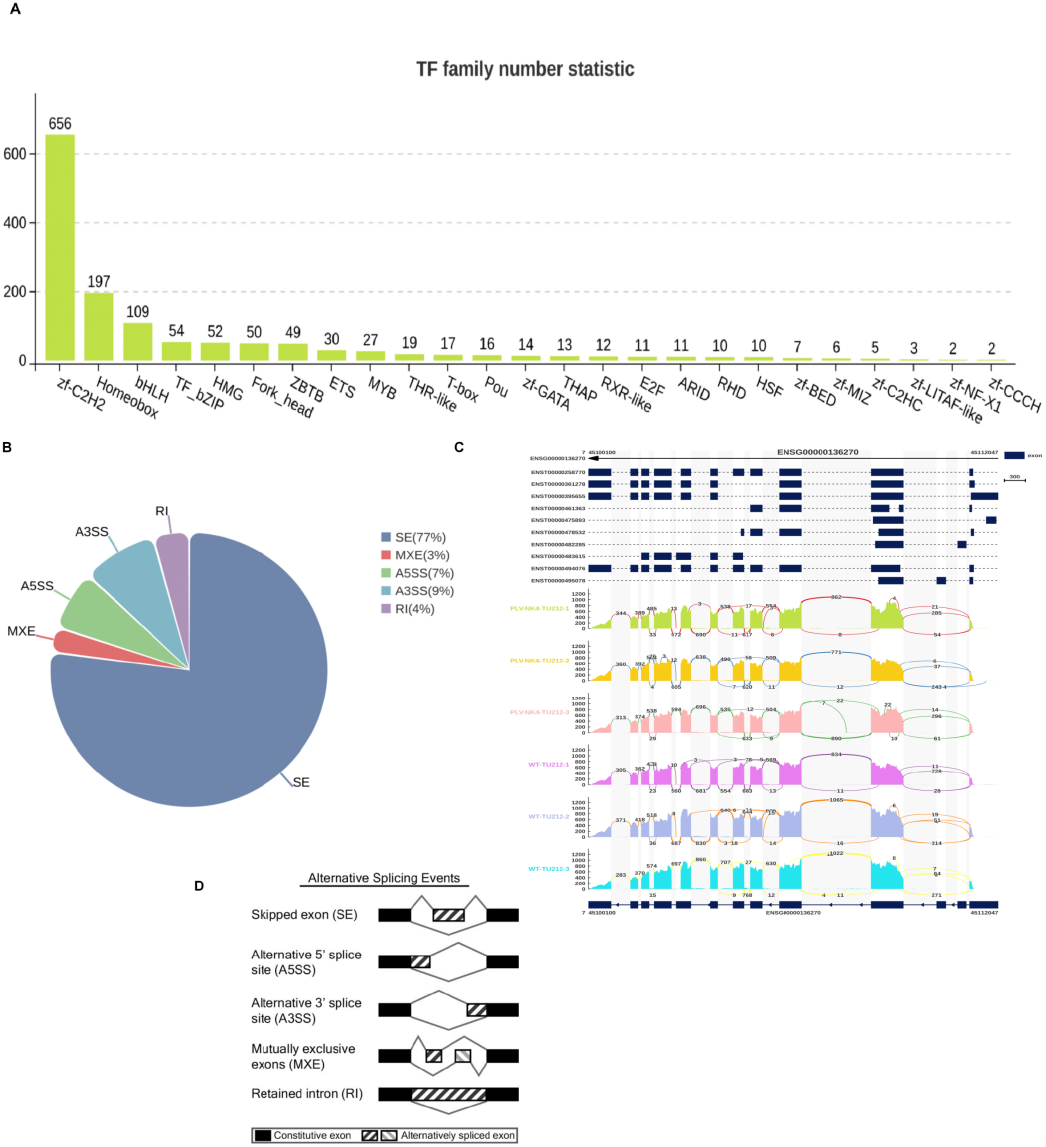
Transcription factor statistics and variable shear analysis (**A–D**).

Transcription factor statistics are mainly focused on zf-C2H2 (**Figure 6A**), and alternative splicing (AS) is an important gene regulatory mechanism in eukaryotes. rMATS is used to detect five types of alternative splicing events (SE, RI, MXE, A5SS, and A3SS), and it can perform differential AS analysis on samples with biological replicates. The analysis results show that exon skipping accounts for the highest proportion (**Figure 6B**).

## 4 Discussion

Laryngeal cancer is a malignant tumor originating in the laryngeal tissues of the larynx (21). Clinical manifestations include progressive hoarseness, a sensation of a foreign body in the throat, difficulty breathing, difficulty swallowing, and enlargement of cervical lymph nodes. Risk factors include long-term smoking, excessive drinking, and human papillomavirus (HPV) infection (22). Treatment for throat cancer emphasizes a balance between organ function preservation and tumor control (23). However, the hidden nature of the disease often leads to a delayed diagnosis (24). Long-term throat edema caused by radiotherapy affects airway function (25). Although research on gene expression markers (such as TP53 and PIK3CA mutations) and immunotherapy targets (such as PD-L1) provides a direction for precise treatment, unified testing standards for clinical applications are lacking (25). Therefore, research on the utilization of gene expression profiles combined with molecular typing and artificial intelligence data analysis to promote personalized and advanced treatment optimization is of great significance (25, 26).

HGF/c-MET signaling is involved in the metabolic reprogramming of tumor cells in head and neck squamous cell carcinoma regulating cell proliferation and invasion capabilities and affecting immune surveillance and activation in the tumor microenvironment (27, 28). NK4 is an intramolecular fragment of HGF that binds to MET but does not activate receptor signaling (29). Previous studies have shown that NK4 adenovirus (Ad-NK4) effectively inhibits the viability, invasiveness, and tumorigenicity of human mesothelioma cells. Additionally, Ad-NK4 suppressed the characteristics of cancer stem cells (30). Human placenta-derived mesenchymal stem cells (PDMSCs) combined with *NK4* inhibit the proliferation ability of glioblastoma cells (15). Heightened HGF expression in head and neck squamous cell carcinomas correlates negatively with patient survival(4). Identification of prognostic genes in the oral squamous cell carcinoma (OSCC) microenvironment revealed that HGF expression significantly correlates with the infiltration levels of B cells, CD4⁺T cells, CD8⁺T cells, macrophages, and neutrophils (31). Blood tests conducted on grouped clinical patients showed that serum HGF concentrations in patients with poorly differentiated tumors, high tumor grades, and advanced clinical stages were significantly higher than those in patients with well-differentiated tumors, low tumor grades, and early clinical stages (p < 0.05) (32). In the present study, we successfully constructed lentiviral vectors carrying FLAG-tagged NK4 overexpression and negative control plasmids. These were successfully packaged into lentiviruses and used to infect the laryngeal squamous cell carcinoma TU212 cell line resulting in the establishment of stable *NK4* gene-expressing TU212 cell lines, PLV-NK4-TU212 and PLV-NC-TU212. The stable transfection of the *NK4* gene in these cells overcomes the drawbacks of low transfection efficiency and unstable gene expression, thus providing an effective and convenient tool for further research. This study validated the effects of the stable transfected cell lines on migration, proliferation, and apoptosis induction capabilities through MTT assays and immunoblotting to detect MET and apoptotic molecular markers. Further validation of the experimental conclusions by Zhang et al. showed that *NK4* gene overexpression can inhibit the proliferation, migration, and invasion abilities of the laryngeal squamous cell carcinoma cell line AMC-HN-8 inducing its apoptosis. *NK4* not only negatively regulates the HGF/c-Met signaling pathway but also inhibits tumor angiogenesis independently of the HGF/c-Met pathway. Studies show that *NK4* inhibits VEGF-induced angiogenesis by suppressing the phosphorylation of ERK and ETS-1 in cultured endothelial cells and in an *in vivo* rabbit model (33), and the anti-angiogenic effect of *NK4* is independent of c-Met. When *NK4* is combined with globin, it inhibits the extracellular assembly of fibronectin, thereby suppressing the spread of fibronectin-dependent endothelial cells (34). *NK4* inhibits the paracrine loop of HGF, indirectly suppressing the expression of VEGF in tumor cells, thereby exerting an anti-angiogenic effect (35). RNA sequencing is an important technique for analyzing differential gene expression in tumors. By analyzing the expression of differential genes, key genes related to specific biological processes can be identified. Total RNA was extracted from PLV-NK4-TU212 and PLV-NC-TU212 cells followed by library construction and sequencing using an Illumina platform. The sequencing results revealed that NK4 caused significant differences in the expression levels of 320 genes, with 189 upregulated and 131 downregulated genes (p < 0.05). Gene Ontology enrichment analysis showed that the distribution of differential genes was primarily focused on biological processes, while Kyoto Encyclopedia of Genes and Genomes pathway enrichment indicated that the pathways involved were mainly related to the HIF-1, MAPK, and PI3K-Akt signaling pathways. This suggests that the expression of the NK4 protein triggered a strong cellular response.

The results obtained from experiments on laryngeal cancer cell lines in this study are consistent with those of previous reports on the anticancer effects of NK4 cells. This suggests that *NK4* could serve as a potential anticancer gene and play an important role in counteracting the growth, invasion, and metastasis of laryngeal cancers.

Due to time constraints, this study did not conduct *in vivo* experimental validation or more in-depth pathway screening and mechanism verification.

## Data Availability

The datasets presented in this study can be found in online repositories. The names of the repository/repositories and accession number(s) can be found in the article/**Supplementary Material**.
