# Correction: Overexpression of NK4 gene in TU212 affects migratory activity in laryngeal squamous cell carcinoma

**DOI:** 10.3389/fonc.2026.1725155

**Published:** 2026-03-13

**Authors:** Yixuan Huo, Wei Zhang, Fan Yang, Wenhua Shao, Guozheng Cong, Shoukai Zhang

**Affiliations:** 1 First Clinical Medical College, Ningxia Medical University, Yinchuan, China; 2 Lanzhou University, Lanzhou, Gansu, China; 3 Lanzhou Veterinary Research Institute, Chinese Academy of Agricultural Sciences, Lanzhou, Gansu, China; 4 Gansu Provincial Hospital, Lanzhou, Gansu, China

**Keywords:** apoptosis, gene expression, laryngeal squamous cell carcinoma, NK4, RNA-seq, migration

There was a mistake in **Figure 2** as published. **Figure 2A**: The PLV-NC-NK4 in the NC group is labeled incorrectly; **Figure 2C**: The OD490nm is labeled incorrectly. The corrected **Figure 2** appears below. “**Figure 2A**: NC group PLV-NC-NK4 changed to PLV-NC-TU212; **Figure 2C**: OD490nm changed to OD570nm”.

**Figure 2:**
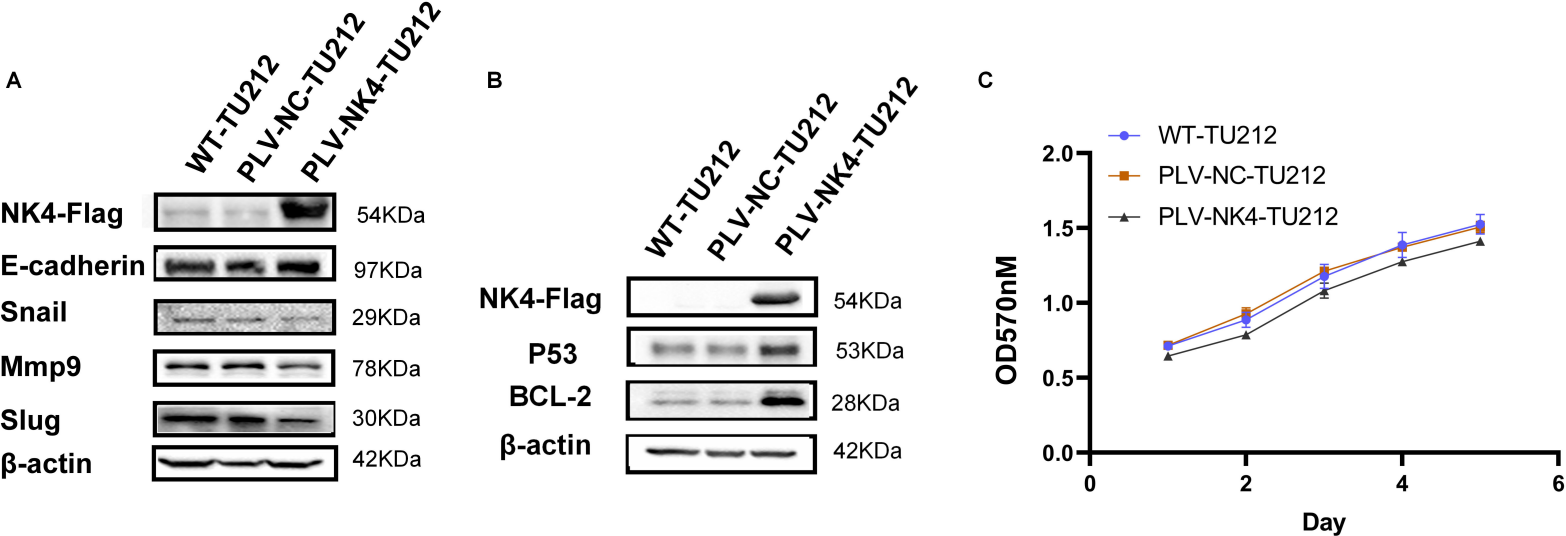
Functional validation of laryngeal squamous cell carcinoma cell lines stably expressing NK4. **(A)** Effect of overexpression of NK4 on TU212 cell migration. **(B)** Effect of NK4 overexpression on apoptosis in TU212 cells. **(C)** Effect of NK4 overexpression on the proliferation ability of TU212.

The funder General projects of the Joint Scientific Research Fund, 24JRRA888 to Shoukai Zhang were erroneously omitted. The correct funding statement reads: "The author(s) declare that financial support was received for the research and/or publication of this article. This work was supported by the Natural Science Foundation of Gansu Province (22JR5RA664) and Joint Scientific Research Fund (24JRRA888) to SK."

There was a mistake in the caption of **Figure 2**, **4**, and **5** as published. Delete the third line (**Figures 4C**) in section 2.4.4. Change (**Figures 4A, B**) to (**Figures 5A, B**) in section 2.5.3. Change (**Figure 5**) to (**Figure 2C**) in section 3.2.2. Change (**Figures 4A, B**) to (**Figures 5A, B**), change (**Figure 4C**) to (**Figure 5C**), change (**Figure 4D**) to (**Figure 5E**) in section 3.2.4. Change (**Figure 4E**) to (**Figure 5G**), and change (**Figure 4F**) to (**Figure 5I**) in section 3.2.5.

A correction has been made to the section 3.2.3 The 4th line was changed from “48h” to “24h”.

A correction has been made to the section 2.4.3, 2.4.4, 2.5.3, 3.2.2, 3.2.3, 3.2.4, 3.2.5:

“2.4.3

A sterile pipette tip was used to create vertical scratches at the bottom of a six-well plate, which had been cultured in complete medium until reaching 100% confluence. The wells were then washed with PBS. Next, RPMI 1640 medium containing 2% serum was added to each well, and the plate was incubated at 37°C with 5% CO₂. Images were captured at 0 and 24 hours using an inverted light microscope. The scratch healing rate was calculated as follows: Scratch healing rate = [(0 h − 24 h) / 0 h] × 100% (**Figure 3**).

2.4.4

Results are expressed as mean ± standard deviation. All data were analyzed using GraphPad Prism 9 (GraphPad Software, Inc., CA). Intergroup differences were assessed using Student’s t-test or unpaired two-tailed t-test. Two-way analysis of variance (ANOVA) was used for comparisons among multiple groups. A p-value <0.05 was considered statistically significant.

2.5.3

with the following conditions for difference screening: p-value < 0.05, |log_2_FC| > log_2_ (1.5) (**Figures 5A, B**).

3.2.2

and the OD value of the PLV-NK4-TU212 group was significantly reduced compared with that of the PLV-NC-TU212 group (**Figure 2C**).

3.2.3

compared to the PLV-NC-TU212 and WT-TU212 groups, the cell migration rate in the PLV-NK4-TU212 group decreased at 24 h suggesting that NK4 overexpression can inhibit the migration ability of TU212 cells

3.2.4

We used RNA-seq to compare the gene expression profiles between PLV-NK4-TU212 and WT-TU212 cell lines, and the results showed that a total of 320 genes had significant differences in expression, of which 189 genes were upregulated and 131 genes were downregulated (**Figures 5A, B**). GO annotations were enriched in terms of molecular function, cellular component, and biological process. In the biological process, we mainly focused on cellular process, biological regulation, regulation of biological process, metabolic process, and response to stimulus; in the cellular component, we only focused on cellular anatomical entity and protein. anatomical entity and protein-containing complex. In terms of molecular function, binding and catalytic activity were differentially enriched (**Figure 5C**). KEGG enrichment analysis showed that among the top 20 significantly enriched pathways, they were mainly related to HIF-1, MAPK, and PI3K–AKt signaling pathways (**Figure 5E**).

3.2.5

The significant enrichment of organ system cancer and cell type cancer in the Disease Ontology (DO) enrichment analysis of differentially expressed genes suggests a dual regulatory mechanism of differentially expressed genes in cancer occurrence (**Figure 5G**). Differentially expressed genes are enriched in the hemostasis pathway, and their abnormalities are closely related to the process of tumor metastasis (**Figure 5I**).”

The original version of this article has been updated.

